# Uncrossed corticospinal tracts in a patient with ichthyosis and hemiparesis: a case report

**DOI:** 10.1186/s12883-020-01698-0

**Published:** 2020-04-06

**Authors:** Huijia Yang, Hongwei Zhou, Jing Miao

**Affiliations:** 1 Department of Neurology and Neuroscience Center, The First Hospital of Jilin University, Changchun, 130021 China; 2 Department of Radiology, The First Hospital of Jilin University, Changchun, 130021 China

**Keywords:** Uncrossed corticospinal tracts, Ihthyosis, DTI, Ischemic stroke, Ipsilateral hemiparesis

## Abstract

**Background:**

Anomalies of pyramidal tract decussation are rare phenomena that can be caused by ectodermal dysplasia. Herein, we describe a patient with ichthyosis who exhibited ipsilateral hemiparesis after stroke and whose neuroimaging results showed evidence of motor control being provided by the ipsilateral motor cortex.

**Case presentation:**

A 24-year-old right-handed man presented with skin abnormalities, sudden-onset left hemiparesis, and dysarthria. He exhibited a mild-to-moderate left-sided weakness (grade 4 on the Medical Research Council scale). Magnetic resonance imaging revealed an acute infarct in the left corona radiata. Diffusion tensor imaging revealed uncrossed corticospinal tracts. Next-generation sequencing identified heterozygous FLG mutations. The patient was diagnosed with cerebral infarction and ichthyosis vulgaris and was treated with aspirin (100 mg/d). His symptoms gradually dissipated.

**Conclusions:**

This case suggests that pyramidal decussation anomalies can be associated with ichthyosis. Patients with ichthyosis should therefore be evaluated for nerve involvement.

## Background

Supratentorial stroke is widely known to cause neurological impairment on the contralateral side of the body. Previous neuroanatomic studies have shown that the primary motor cortex predominantly controls the contralateral half of the body [1]. Corticospinal tract decussation anomalies are mostly nonspecific and coincidental. Since the first report [2], such anomalies have been described in patients with posterior fossa malformations and extensive brainstem malformations [3]. Furthermore, a few reports have described ipsilateral hemiparesis in patients with agenesis of the corpus callosum and scoliosis [4, 5], and such reports show that pyramidal tract abnormalities can arise in patients with developmental disorders. Herein, we describe a patient with ichthyosis who exhibited ipsilateral hemiparesis after stroke and whose neuroimaging results showed evidence of motor control being provided by the ipsilateral motor cortex.

## Case presentation

**Fig. 1:**
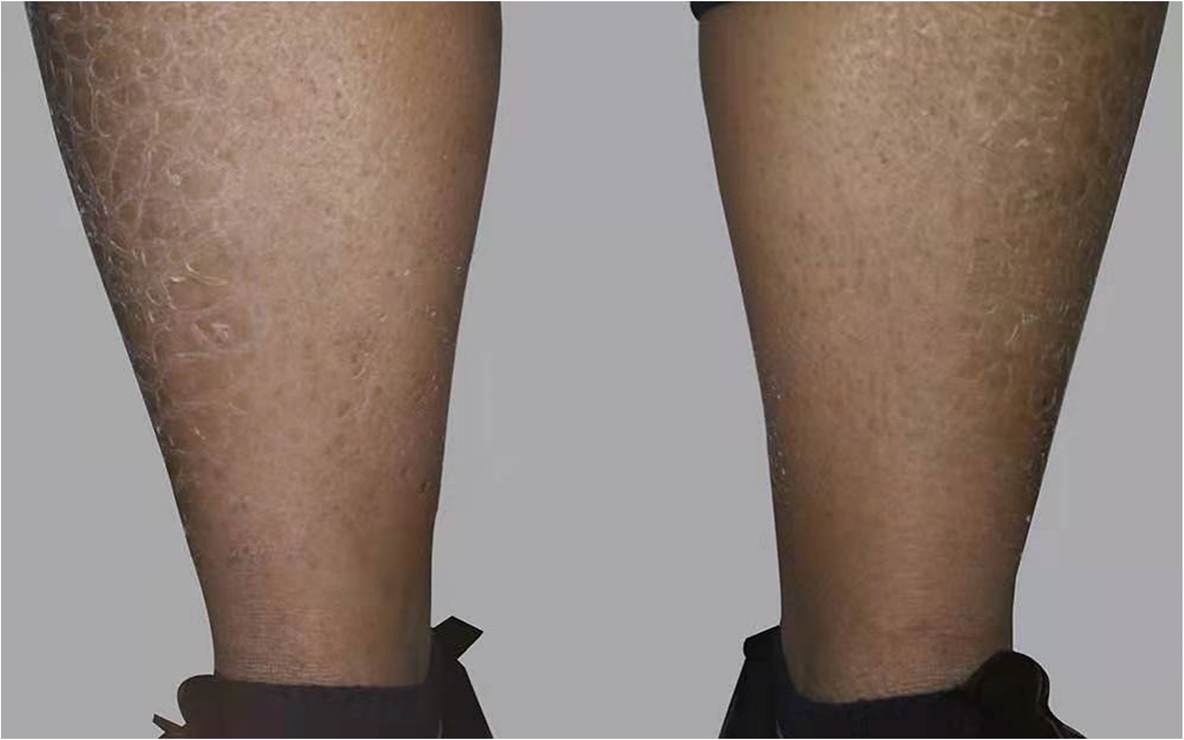
The skin on the patient’s shins was dry and scaly

A 24-year-old man presented with sudden-onset mild left-sided hemiparesis that had manifested 2 days earlier. He had no history of smoking, or excessive alcohol consumption. A medical examination showed that he had dry and scaly skin (Fig. 1). He claimed to have experienced his skin disorder for 20 years. On neurological examination, he was conscious and alert. No other focal neurologic deficits were noted except mild left-sided hemiparesis.

**Fig. 2:**
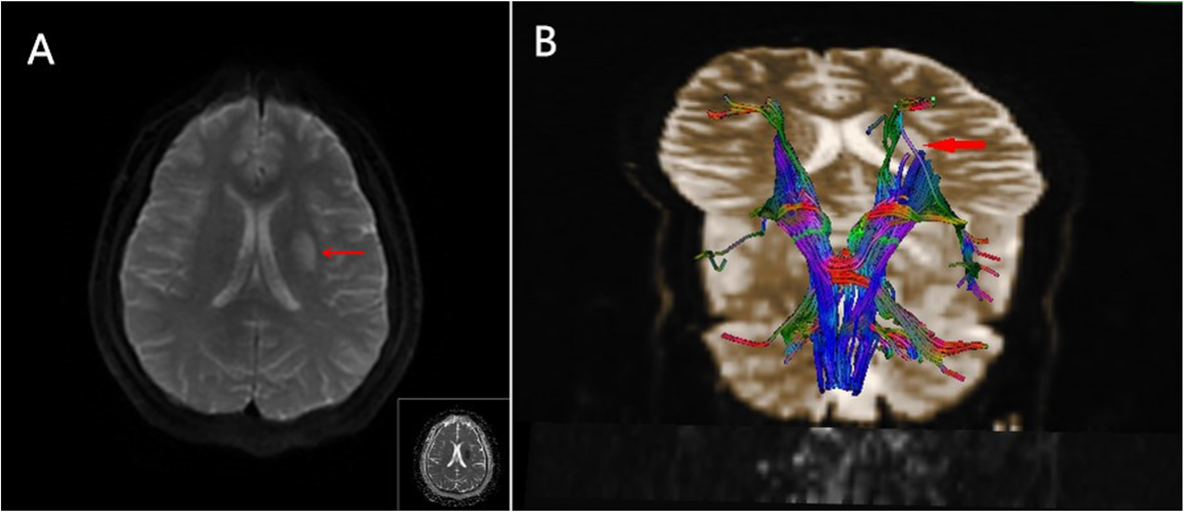
**a**. Diffusion-weighted imaging revealed lesions with strong signal intensities and decreased apparent diffusion coefficients. **b**. Diffusion tensor imaging tractography revealed uncrossed corticospinal tracts. The red arrow shows the infarcted area in which the corticospinal tract was destroyed

Initial head CT demonstrated no acute infarction. The following day, brain magnetic resonance imaging (MRI) (Siemens, Germany) with diffusion-weighted sequences revealed punctate areas of restricted diffusion in the left corona radiata that represented small acute infarct (Fig. 2a). Magnetic resonance angiography revealed no significant flow-limiting stenosis. Because the MRI-detected lesions were ipsilateral to the paralyzed limb, the patient underwent diffusion tensor imaging (DTI) (Siemens Magnetom trioatim 3.0 T, Siemens Leonardo) tractography, which revealed uncrossed corticospinal tracts (Fig. 2b). The level of D-dimer was within the normal range in our patient who without history of hypertensive disease, in addition, no abnormalities were found in carotid ultrasound, cervical MRI, echocardiography, foaming test and microembolism monitoring, the probabilities of arterial dissection or patent foramen ovale were reduced.

Cerebrovascular risk factors such as serum lipid, uric acid, homocystein, high-sensitivity C-reactive protein, B12 vitamin, folate, fasting blood glucose were all within the normal range and the patient has no history of hypertension, diabetes mellitus, smoking, and alcohol consumption. Body mass index (BMI), routine blood test, coagulation routine, renal function and liver function, erythrocyte sedimentation rates (ESR), HIV serology, syphilis serology, serum lactate, anti-nuclear antibody were all normal or negative. An electrocardiogram and a transthoracic echocardiogram revealed no sources of cardiac embolism. Besides, the patient’s other MRI sequences were negative, so we could exclude cerebral small vessel disease and microbleeds. Noteworthy, his skin was abnormal for about 20 years. We performed a genetic test on the patient. However, next-generation sequencing of a DNA sample identified two known heterozygous FLG nonsense mutations: (c.3905C > A, p.Ser1302Ter)(which was inherited from his father) and (c.5368C > T, p.Gln1790Ter) (which was inherited from his mother) [6, 7]. The two mutations have an effect on the protein functions.

Based on these genetic findings and the patient’s 20-year history of skin complaints, the patient was diagnosed with ichthyosis vulgaris and cerebral infarction. His left-side hemiparesis gradually dissipated after symptomatic treatment. Aspirin therapy played a crucial role in the secondary prevention of ischemic stroke. After discharge, the patient continued to take aspirin with daily dose of 100 mg.

## Discussion

Ichthyosis is a group of disorders resulting from variants in more than 50 genes, and it is characterized by thickening and splitting of the skin [8]. Roy et al. [9] reported that spasticity, epilepsy, and intellectual disability are the primary neurological findings. Our case represents the first report of cerebral infarctions as neurological comorbidities in a patient with ichthyosis.

We diagnosed our patient with ichthyosis vulgaris, which typically results from heterozygous or compound heterozygous *FLG* mutations. However, all forms of ichthyosis have been associated with protein and lipid abnormalities [8]. Our young patient had almost no risk factors for cerebrovascular disease, indicating that ichthyosis might be associated with the development of infarction. Interestingly, our patient exhibited brain lesions that were ipsilateral to his paralyzed limb.

Previous reports suggested anomalies of the dorsal column medial lemniscus pathway associated with uncrossed pyramidal tracts and abnormalities of bimanual tasks in pyramidal tract crossing anomalies [10–12]. After we had performed a careful physical examination and asked history of disease entirely, not found any sensory abnormality, synkinetic contralateral movements or mirror movement in this patient.

Donkelaar et al. [3] described the development and potential malformations of the human pyramidal tract and noted that certain malformations may lead to pyramidal decussation anomalies. Decussation anomalies of the corticospinal tracts can be found in association with other cerebral anomalies, such as congenital scoliosis, corpus callosum agenesis, occipital encephaloceles, posterior fossa malformations, Dandy-Walker malformations, Joubert syndrome, and extensive brainstem malformations [3].

In terms of embryogenesis, the skin and nervous system are homologous to the ectoderm. Congenital ectodermal dysplasia causes simultaneous neurological and cutaneous lesions, resulting in various clinically complex hereditary neurocutaneous syndromes. Sprecher et al. [13] described a novel neurocutaneous disease called CEDNIK syndrome that is characterized by cerebral dysgenesis, neuropathy, ichthyosis, and keratoderma. We therefore speculate that our patient’s pyramidal decussation anomalies may be caused by the same FLG mutations that cause his ichthyosis.

## Conclusions

In conclusion, we identified uncrossed pyramidal tracts in a patient with clinically and genetically confirmed ichthyosis and there might be a pathophysiological association, becoming clinically relevant in certain scenarios, such as stroke.

## Abbreviations

CT: Computed tomography
MRI: Magnetic resonance imaging
DTI: Diffusion tensor imaging
